# Isolation and functional characterization of CE1 binding proteins

**DOI:** 10.1186/1471-2229-10-277

**Published:** 2010-12-16

**Authors:** Sun-ji Lee, Ji Hye Park, Mi Hun Lee, Ji-hyun Yu, Soo Young Kim

**Affiliations:** 1 Department of Molecular Biotechnology and Kumho Life Science Laboratory, College of Agriculture and Life Sciences, Chonnam National University, Gwangju 500-757, South Korea

## Abstract

**Background:**

Abscisic acid (ABA) is a plant hormone that controls seed germination, protective responses to various abiotic stresses and seed maturation. The ABA-dependent processes entail changes in gene expression. Numerous genes are regulated by ABA, and promoter analyses of the genes revealed that *cis*-elements sharing the ACGTGGC consensus sequence are ubiquitous among ABA-regulated gene promoters. The importance of the core sequence, which is generally known as ABA response element (ABRE), has been demonstrated by various experiments, and its cognate transcription factors known as ABFs/AREBs have been identified. Although necessary, ABRE alone is not sufficient, and another *cis*-element known as "coupling element (CE)" is required for full range ABA-regulation of gene expression. Several CEs are known. However, despite their importance, the cognate transcription factors mediating ABA response via CEs have not been reported to date. Here, we report the isolation of transcription factors that bind one of the coupling elements, CE1.

**Results:**

To isolate CE1 binding proteins, we carried out yeast one-hybrid screens. Reporter genes containing a trimer of the CE1 element were prepared and introduced into a yeast strain. The yeast was transformed with library DNA that represents RNA isolated from ABA-treated Arabidopsis seedlings. From the screen of 3.6 million yeast transformants, we isolated 78 positive clones. Analysis of the clones revealed that a group of AP2/ERF domain proteins binds the CE1 element. We investigated their expression patterns and analyzed their overexpression lines to investigate the *in vivo *functions of the CE element binding factors (CEBFs). Here, we show that one of the CEBFs, AtERF13, confers ABA hypersensitivity in Arabidopsis, whereas two other CEBFs enhance sugar sensitivity.

**Conclusions:**

Our results indicate that a group of AP2/ERF superfamily proteins interacts with CE1. Several CEBFs are known to mediate defense or abiotic stress response, but the physiological functions of other CEBFs remain to be determined. Our *in vivo *functional analysis of several CEBFs suggests that they are likely to be involved in ABA and/or sugar response. Together with previous results reported by others, our current data raise an interesting possibility that the coupling element CE1 may function not only as an ABRE but also as an element mediating biotic and abiotic stress responses.

## Background

Abscisic acid (ABA) is a phytohormone that controls seed germination, seedling growth and seed development [1]. In particular, ABA plays an essential role in the protective responses of plants to adverse environmental conditions, such as drought, high salinity and extreme temperatures [2].

At the molecular level, ABA-dependent processes entail changes in gene expression patterns. Numerous genes are either up- or down-regulated by ABA in seedlings [3,4]. The ABA regulation of these genes is generally at the transcriptional level, and a number of *cis*-regulatory elements responsible for the regulation by ABA have been determined [5]. One of the *cis*-elements consists of ACGTGGC core sequence. The element, which is similar to the G-box (CACGTG) present in many light-regulated promoters [6], is ubiquitous among ABA-regulated gene promoters and generally known as ABA response element (ABRE). Although necessary, a single copy of the G-box type ABRE is not sufficient to mediate ABA regulation, and multiple copies of ABRE or combinations of ABRE with another *cis*-element are required for the full ABA-induction of genes. For instance, an element known as CE3 (coupling element 3, ACGCGTGTCCTC) is required for the ABA-induction of barley *HVA1 *and *OsEm *genes [7]. Thus, CE3 and ABRE constitute an ABA response complex. Another coupling element, CE1 (TGCCACCGG), is necessary for the ABA-regulation of *HVA22 *gene [8]. In *RD29A *gene, DRE (Dehydration-responsive element, TACCGACAT) functions as a coupling element to ABRE [9].

A subfamily of bZIP proteins has been identified that mediate the ABA response via the G-box type ABRE in Arabidopsis [10,11]. Referred to as ABFs or AREBs, these proteins not only bind the ABRE but also mediate stress-responsive ABA regulation in Arabidopsis seedlings [12]. On the other hand, ABI5, which belongs to the same subfamily of bZIP proteins as ABFs/AREBs [13,14], mediates ABA response in the embryo. ABFs/AREBs were isolated based on their binding to ABRE. Subsequent study showed that they also bind the coupling element CE3 [10], which is functionally equivalent to ABRE [15].

The transcription factors that bind the CE1 element have not been reported yet. Among the known transcription factors involved in ABA response, ABI4 has been shown to bind the CE1 element [16]. However, the preferred binding site of ABI4 is CACCG, which differs from the CE1 element consensus CCACC. Thus, it has been suggested that AP2 domain proteins other than ABI4 would interact with CE1 [17].

To isolate CE1 element binding factors, we conducted yeast one-hybrid screens. From the screen of 3.6 million yeast transformants, we isolated 78 positive clones. Analysis of the clones revealed that a group of AP2/ERF domain proteins bind the CE1 element in yeast. Most of the CE1 binding factors (CEBFs) belong to the B-3 or the A-6 subfamily of AP2/ERF domain proteins [18,19]. We also found that overexpression of some of the CEBFs alters ABA and/or sugar responses in Arabidopsis.

## Results

### Isolation of CE1-binding proteins

To isolate genes encoding the proteins that bind the CE1 element, we conducted yeast one-hybrid screens [10]. A trimer of the CE1 element was cloned in front of the minimal promoters of the *lac*Z and the *HIS3 *reporter genes, respectively. The reporter constructs were then introduced into a yeast strain to create reporter yeast, which was subsequently transformed with cDNA library DNA. The library was prepared from mRNA isolated from ABA-and salt-treated Arabidopsis seedlings. The resulting transformants were screened for reporter activities. From the screen of 3.6 million yeast transformants, we obtained 78 positive clones and analyzed more than 50 clones.

Grouping of the positive clones based on their insert restriction patterns and subsequent DNA sequencing revealed that they encode a group of AP2/ERF superfamily transcription factors (Table 1). Twelve isolates encoded AtERF15 (At2g31230), ten isolates encoded ERF1 (At3g23240) and nine isolates encoded RAP2.4 (At1g78080). Other multiple or single isolate encoded AtERF1 (At4g17500), AtERF5 (At5g47230), AtERF13 (At2g44840) and seven other AP2/ERF family proteins. Among the 13 AP2/ERF proteins isolated, nine belong to the B-3 subfamily, three belong to the A-6 subfamily and one belongs to the B-2 subfamily. Thus, a group of AP2/ERF proteins, especially those belonging to the subgroup B-3, was isolated as CE1-binding factors in our one-hybrid screen. We designated the proteins CEBFs (CE1 binding factors).

**Table 1:** Results of one-hybrid screen: CE1 element binding factors (CEBFs)

No. isolates	Gene ID	Gene name	Conserved domain	Group^a^
12	At2g31230	AtERF15	AP2/ERF	B-3 subfamily
10	At3g23240	ERF1	"	B-3 subfamily
4	At4g17500	AtERF1	"	B-3 subfamily
5	At5g47230	AtERF5	"	B-3 subfamily
2	At2g44840	AtERF13	"	B-3 subfamily
1	At5g47220	AtERF2	"	B-3 subfamily
1	At5g07580	--	"	B-3 subfamily
1	At1g06160	ORA59	"	B-3 subfamily
1	At5g61590	--	"	B-3 subfamily
2	At1g53910	RAP2.12	"	B-2 subfamily

9	At1g78080	RAP2.4	"	A-6 subfamily
2	At1g22190	RAP2.4L	"	A-6 subfamily
2	At4g13620	--	"	A-6 subfamily

^a ^Grouping is according to Sakuma et al. [19].

### DNA-binding and transcriptional activities of CEBFs

**Figure 1:**
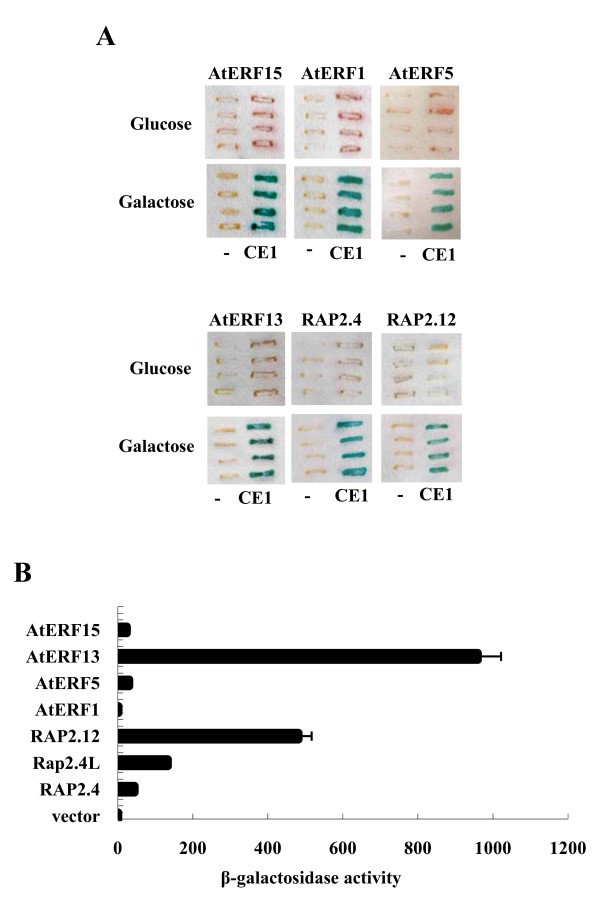
**Binding and transcriptional activities of CEBFs**. (A) Binding of CEBFs to the CE1 element. The binding activity of six CEBFs was confirmed in yeast. Reporter yeast containing the *lacZ *reporter gene with (CE1) or without (-) the CE1 element was transformed with DNA from positive clones, and the transformants were grown in the glucose- or galactose-containing medium and assayed for the β-galactosidase activity by filter lift assay. (B) Transcriptional activity of CEBFs. CEBFs were cloned in frame with *Lex*A DB, the fusion constructs were introduced into reporter yeast containing the *lac*Z reporter, and the reporter activity was measured by a liquid β-galactosidase assay. The numbers indicate the enzyme activity in Miller units. Each data point represents the mean of four independent measurements, and the small bars indicate the standard errors.

Binding of a number of CEBFs, which were isolated as multiple isolates (Table 1), to the CE1 element was confirmed in yeast. Plasmid DNA was isolated from the positive clones, and their binding to CE1 was determined by investigating their ability to activate the CE1-containing *lac*Z reporter gene. Figure 1A shows the results obtained with six different positive clones: AtERF15, AtERF5, AtERF1, AtERF13, RAP2.4 and RAP2.12. The four AtERFs, which belong to the B-3 subfamily, could activate the reporter gene containing the CE1 element but not the reporter gene lacking the CE1 element. The CE1-dependent reporter activation was observed with medium containing galactose but not with the medium containing glucose. Thus, reporter activation was also dependent on the presence of galactose, which is an inducer of the GAL1 promoter that drives the expression of the cDNA clones. Similarly, RAP2.12 and RAP2.4, which belong to the B-2 and the A-6 subfamily, respectively, could also activate the reporter gene, and the activation was CE1- and galactose-dependent.

CEBFs are putative transcription factors; accordingly, we wanted to determine if they possess transcriptional activity. To accomplish this, the transcriptional activity of CEBFs was examined employing a yeast assay system. The coding regions of seven CEBFs were individually cloned in frame with the *Lex*A DB in the vector pPC62LexA [20]. The hybrid constructs were then introduced into the yeast strain L40, which carries a *lac*Z reporter gene with an upstream *Lex*A operator in its promoter. Figure 1B shows that AtERF13 has the highest transcriptional activity among the seven CEBFs. RAP2.12 also possesses high transcriptional activity, while RAP2.4, RAP2.4L (At1g22190), AtERF5 and AtERF15 displayed relatively lower transcriptional activity. AtERF1 was found to have very low transcriptional activity.

### Expression patterns of CEBFs

**Figure 2:**
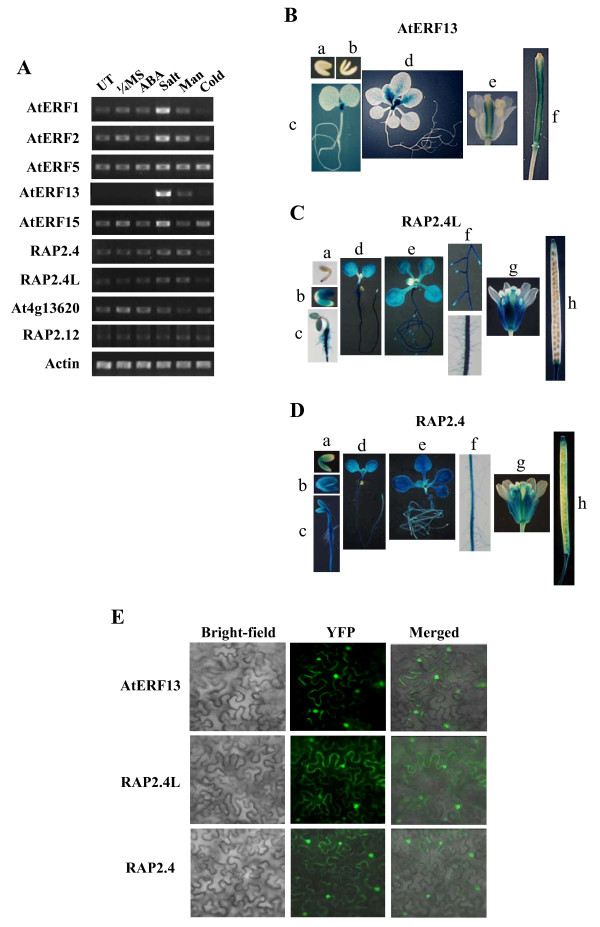
**Expression patterns of CEBFs**. (A) Induction patterns of CEBFs were determined by RT-PCR. UT, untreated plants. Plants were treated with 1/4MS, ABA, NaCl (Salt), 600 mM mannitol (Man) for 4 hrs, or placed at 4 C for 24 hr (Cold) before RNA was isolated. (B) Histochemical GUS staining of transgenic plants harboring AtERF13 promoter-GUS reporter gene construct. a. immature embryo. b. mature embryo. c, 5-day-old seedling. d, 15-day-old seedling. e, flower. f, immature silique. (C) Histochemical GUS staining of transgenic plants harboring the RAP2.4L promoter-GUS reporter gene construct. a. mature embryo. b. mature embryo. c, 2-day-old seedling. d, 5-day-old-seedling. e, 14-day-old seedling. f, roots of 14-day-old seedling. g, flower. h, mature silique. (D) Histochemical GUS staining of transgenic plants harboring RAP2.4 promoter-GUS reporter gene construct. a. mature embryo. b. mature embryo. c, 2-day-old seedling. d, 5-day-old-seedling. e, 14-day-old seedling. f, roots of 14-day-old seedling. g, flower. h, mature silique. In B-D, GUS staining was conducted for 20 hrs. (E) Subcellular localization of AtERF13, RAP2.4L and RAP2.4. Tobacco leaves were infiltrated with *Agrobacterium *as described in the Methods and observed with fluorescence microscope 40 hrs after infiltration. Bright field, fluorescence (YFP) and merged images of the tobacco leaves are shown.

The expression patterns of nine CEBFs in seedlings were examined by coupled reverse transcription and polymerase chain reaction (RT-PCR). Because the tissue-specific expression patterns of many AP2/ERF domain proteins have been reported [21], we focused on the ABA and stress induction patterns of CEBFs. Figure 2A shows that the expression of AtERF1, AtERF2, AtERF13 and AtERF15 was induced by high salt. In the case of AtERF13, its expression was also induced by high osmolarity (i.e., mannitol). The expression of other CEBFs was largely constitutive or their induction levels were very low.

For AtERF13, RAP2.4 and At1g22190, which was designated RAP2.4L (RAP2.4-like) because of its high similarity to RAP2.4, we examined their tissue-specific expression patterns in detail by investigating their promoter activity. Transgenic plants harboring the promoter-GUS reporter constructs were prepared, and histochemical GUS staining was carried out to determine their temporal and spatial expression patterns.

With ATERF13, GUS activity was observed only in the shoot meristemic region and the emerging young leaves in seedlings (Figure 2B). Thus, AtERF13 expression in seedlings was specific to the shoot meristem region. During the reproductive stage, GUS activity was observed in the carpels. On the other hand, GUS activity was observed in most of the tissues with the RAP2.4L promoter (Figure 2C). GUS activity was not observed in the immature embryo, but it is detected in the mature embryo and most of the seedling tissues. The GUS activity was strong in most of the tissues, although relatively weaker activity was observed in young leaves and the lateral root tips including the meristem and the elongation zone. Strong GUS activity was also observed in reproductive organs such as sepals, filaments, style and abscission zone. The GUS staining pattern of the transgenic plants harboring the RAP2.4 promoter construct was similar to that of the plants harboring the RAP2.4L promoter construct (Figure 2D). In general, stronger GUS activity was observed with the RAP2.4 promoter, and, unlike the RAP2.4L promoter, its activity was detected in the emerging young leaves.

To obtain further clues to the function of AtERF13, RAP2.4L and RAP2.4, we determined their subcellular localization. The coding regions of the CEBFs were individually fused to EYFP under the control of the 35 S promoter, and the localization of the fusion proteins was examined after Agroinfiltration of tobacco leaves. Figure 2E shows that YFP signal is detected in the nucleus with the AtERF13 construct. Similarly, the YFP signal was also observed in the nucleus with RAP2.4L and RAP2.4. Thus, our results indicate that AtERF13, RAP2.4L and RAP2.4 are localized in the nucleus.

### *In vivo *functions of CEBFs

Our transcriptional assay (Figure 1B) showed that AtERF13 has the highest transcriptional activity among CEBFS, and its expression was highly inducible by high salt (Figure 2A). Hence, we chose AtERF13 for functional analysis. To determine the *in vivo *function of AtERF13, we generated its overexpression (OX) lines. The coding region of AtERF13 was fused to the CaMV 35 S promoter employing the pBI121 vector [22], and after transformation of Arabidopsis, T3 or T4 generation transgenic plants were recovered and used for phenotype analysis.

**Figure 3:**
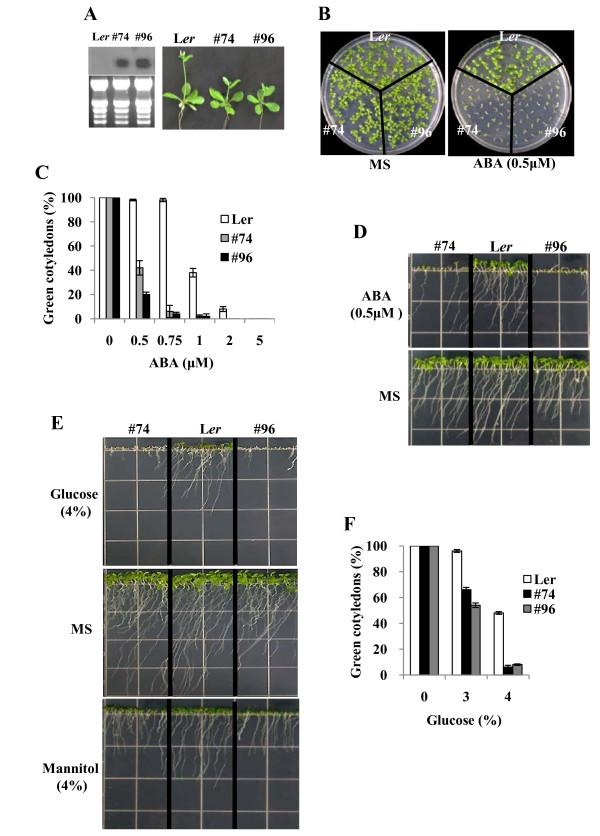
**ABA and glucose sensitivity of AtERF13 overexpression lines**. (A) Growth of AtERF13 OX lines. Three-week-old plants grown in soil. The numbers indicate line no. and the left panel shows the AtERF13 expression levels determined by Northern analysis. (B) Growth of the OX lines in the presence of 0.5 μM ABA. Seeds were germinated and grown for 10 days. (C) ABA dose response of shoot development measured by cotyledon greening efficiency. Seeds were germinated and grown for 11 days on MS medium containing various concentrations of ABA, and seedlings with green cotyledons were counted. Experiments were done in triplicates (n = 50 each), and the small bars indicate standard errors. (D) Root growth of the OX lines in the presence of 0.5 μM ABA. (E) Growth of the OX lines in the presence of 4% glucose. (F) Glucose dose response determined by cotyledon greening. Plants were grown on MS medium containing 3 or 4% glucose for 11 days before counting seedlings with green cotyledons. Experiments were conducted in triplicates (n = 50 each). The small bars represent standard errors.

AtERF13 OX lines exhibited minor growth retardation (Figure 3A), and mature plants were slightly smaller than the wild type plants (not shown). However, other than minor dwarfism, the overall growth pattern was normal. Because the CE1 element is an ABA response element, we determined the ABA-associated phenotypes to address whether AtERF13 overexpression affected ABA response. The germination rates of the transgenic plants were slightly slower (~2hr) in ABA-free medium (not shown), but the ABA sensitivity of the transgenic seed germination was similar to that of the wild type plants (not shown).

Unlike the seed germination, postgermination growth of the AtERF13 OX lines exhibited altered ABA response. Figure 3B and Figure 3C show that shoot development of the transgenic plants was inhibited severely at low concentrations of ABA. For instance, cotyledons of less than 50% of the transgenic plants turned green at 0.5 μM ABA, and true leaf development was not observed with any of the transgenic plants. By contrast, shoot development of wild type seedlings was not affected significantly by the same concentration of ABA. Similarly, root growth of the AtERF13 OX lines was also severely inhibited at 0.5 μM ABA, whereas root growth of the wild type plants was affected much less (Figure 3D). Thus, postgermination growth of the AtERF13 OX lines was hypersensitive to ABA.

We next examined the glucose sensitivity of the AtERF13 OX lines. Glucose inhibits shoot development, i.e., cotyledong greening and true leaf development, and the inhibition process is mediated by ABA [23]. Figure 3E and Figure 3F show that glucose-dependent arrest of shoot development was much more severe in the AtERF13 OX lines. At 3% glucose, cotyledon greening of the wild type plants was not affected noticeably. By contrast, cotyledon greening efficiency of the transgenic plants was reduced to approximately 50%. At 4% glucose, shoot development was observed with approximately 50% of the wild type plants, whereas less than 10% of the OX lines develop green cotyledons. Thus, our results indicated that AtERF13 OX lines are hypersensitive to glucose. We did not observe changes in mannitol (Figure 3E) or salt (Additional file 1) response in parallel experiments, suggesting that the effect is glucose-specific.

**Figure 4:**
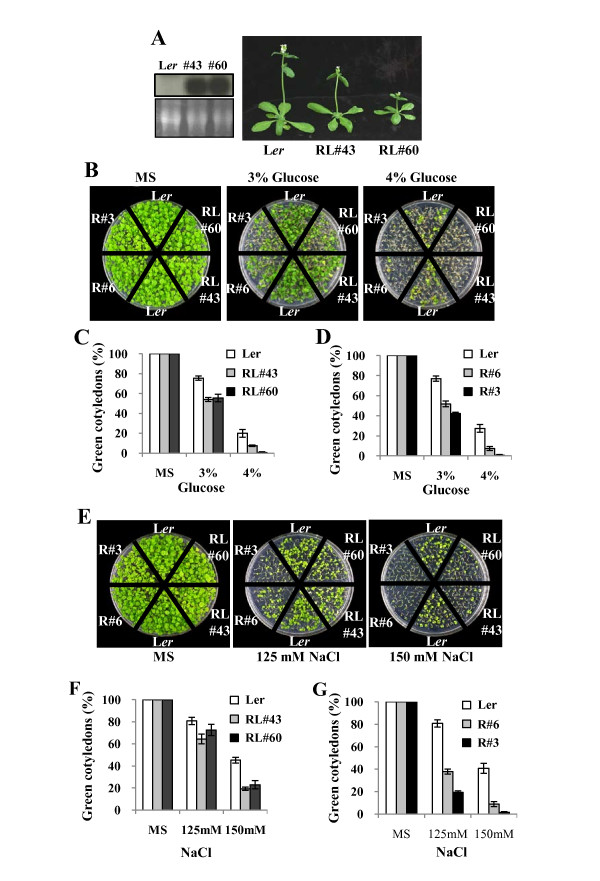
**Glucose and salt sensitivity of RAP2.4L overexpression lines**. (A) Growth of RAP2.4L OX lines. Plants were grown in soil for five weeks. The left panel shows the RAP2.4L expression levels in the transgenic lines (#43 and #60) determined by Northern analysis. (B) Plants grown in the presence of 3% or 4% glucose for 13 days. R, RAP2.4. RL, RAP2.4L. The numbers indicate line numbers. (C) Glucose dose response of RAP2.4L OX lines. (D) Glucose dose response of RAP2.4 OX lines. (E) Plants grown in the presence of salt for 13 days. (F) Salt dose response of RAP2.4L OX lines. (G) Salt dose response of RAP2.4 OX lines. In (C), (D), (F) and (G), experiments were conducted in triplicates (n = 45 each), and the small bars indicate the standard errors.

We conducted similar experiments to investigate the *in vivo *function of RAP2.4L, which belongs to the A-6 subfamily and whose function has not been reported yet. RAP2.4L OX lines were constructed, and their phenotypes were scored to address its involvement in ABA response. The transgenic plants displayed minor growth retardation (Figure 4A), but no distinct changes in ABA sensitivity were observed. On the other hand, the RAP2.4L OX lines displayed altered response to glucose. Figure 4B and Figure 4C show that shoot development of the RAP2.4L OX lines was more severely inhibited by 3% and 4% glucose than the wild type plants. As mentioned above, RAP2.4 is highly homologous to RAP2.4L. Therefore, we prepared RAP2.4 OX lines and analyzed their phenotypes as well (see Discussion). We did not observe changes in ABA sensitivity; however, similar to RAP2.4L OX lines, the RAP2.4 OX lines were hypersensitive to glucose (Figure 4B and Figure 4D). We also examined the salt tolerance of RAP2.4L and RAP2.4 OX lines. The results showed that postgermination growth, i.e., cotyledon greening and true leaf development of both transgenic lines was more severely inhibited at 125 and 150 mM NaCl than wild type plants. The salt sensitivity of RAP2.4 OX lines was more pronounced than that of RAP2.4L. We did not observe changes in mannitol sensitivity in either the RAP2.4 or the RAP2.4L OX lines (Additional file 2).

To further confirm their involvements in ABA response, we analyzed knockout lines of RAP2.4L and RAP2.4 and RNAi lines of AtERF13. We did not observe distinct phenotypes with the transgenic plants, presumably because of the functional redundancy among CEBFs.

**Figure 5:**
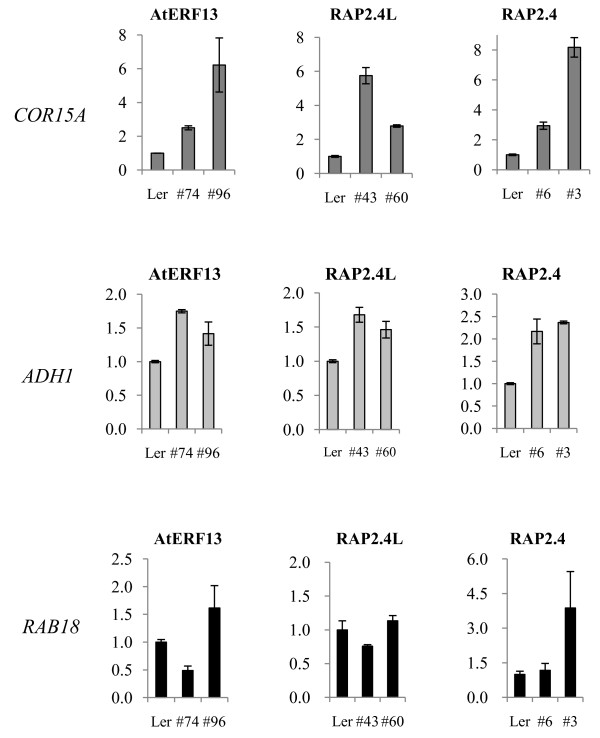
**Expression of ABA-responsive genes in AtERF13, RAP2.4L and RAP2.4 overexpression lines**. Expression of three ABA-regulated genes (*COR15A*, *ADH1 *and *RAB18*) was determined by Real-Time RT-PCR. Reactions were conducted in duplicates, and the small bars indicate the standard errors.

To investigate the target genes of AtERF13, we determined the changes in the expression levels of a number of ABA-responsive genes by Real-Time RT-PCR. Among the genes we investigated, the expression level of *COR15A *increased significantly in the AtERF13 OX lines (Figure 5). Slight increases in *ADH1 *expression were also observed. By contrast, *RAB18 *expression decreased or increased slightly in the transgenic lines. Similar analysis showed that *COR15A *and *ADH1 *expression levels were enhanced in the RAP2.4L and the RAP2.4 OX lines. Increase in the *RAB18 *expression level was also observed in the RAP2.4 OX line (#3). The three genes whose expression levels were altered in the transgenic lines have the G-box type ABREs in their promoter regions and are inducible by both ABA and various abiotic stresses. Additionally, *COR15A *and *RAB18 *genes have a sequence element (i.e., CCGAC) that can function as another coupling element, DRE, although the CE1 core sequence, CCACC, was not found.

## Discussion

We isolated genes encoding CE1 element binding factors (CEBFs) employing a yeast one-hybrid system. CEBFs belong to the AP2/ERF superfamily of transcription factors [18,19]. The AP2/ERF proteins are classified into three families: AP2, ERF and RAV. Whereas AP2 and RAV family members possess an additional AP2 or B3 DNA-binding domain, ERF family members possess a single AP2/ERF domain. The ERF family is further divided into two subgroups, the DREB/CBF subfamily (group A) and the ERF subfamily (group B) [19]. Among the 52 positive clones we analyzed, 39 encoded B group proteins (i.e., B-3 subfamily members), whereas 13 encoded A group proteins (i.e., A-6 subfamily members) (Table 1).

The *in vitro *binding sites of many AP2/ERF superfamily proteins have been studied in detail. The DRE core sequence, i.e., the binding site for DREB1A and DREB2A, which are representative members of the DREB/CBF subfamily, is A/GCCGAC [19]. The GCC box core sequence, which is the consensus binding site for ERF family members, is AGCCGCC [24]. Thus, the two sequences share the CCGNC consensus sequence, the central G being essential for high affinity binding. On the other hand, the core sequence of the CE1 element is CCACC, which differs from the DRE and the GCC box core sequences. The results of our one-hybrid screen indicate that a subset of AP2/ERF family members (i.e., at least ten B-3/B-2 subgroup members and three A-6 subgroup proteins) bind the CE1 element in yeast.

Several of the CEBFs have been reported as GCC box binding proteins. For example, the preferred *in vitro *binding site of AtERF1, AtERF2 and AtERF5 is the wild type GCC box, AGCCGCC [25]. Mutations of the Gs at the second and fifth positions reduced their binding activity to less than 10% of that obtained with the wild type sequence. Similarly, the mutation of the second G of the core sequence greatly reduced the *in vitro *binding of RAP2.4 [26]. However, in our one-hybrid screen, AtERF1, AtERF5 and RAP2.4 were isolated as multiple isolates (i.e., 4, 5 and 9 isolates, respectively). The result suggests that these proteins can interact with the non-GCC box sequence, CCACC, under physiological conditions (i.e. in yeast).

AP2/ERF proteins are involved in various cellular processes, including biotic and abiotic stress responses [18,19]. Many DREB/CBF family proteins (e.g., DREB1A, DREB1B, DREB1C, DREB2A, RAP2.1 and RAP2.4) are involved in ABA-independent abiotic stress responses [19,26,27], whereas ERF family members (e.g., ERF1, ORA59, AtERF2, AtERF4, AtERF14, and RAP2.3) are generally involved in ethylene and pathogen defense responses [18,28-34]. In particular, several of the AP2/ERF proteins are involved in ABA response. ABI4, which belongs to the DREB/CBF subfamily, is a positive regulator of ABA and sugar responses [35]. DREB2C and maize DBF1 are also positive regulators of ABA response [36,37]. On the other hand, AtERF7 [38], ABR1 [39] and AtERF4 [34] are ERF subfamily proteins that are negative regulators of ABA response.

To determine the *in vivo *functions of CEBFs in ABA response, we generated their OX lines and acquired knockout lines for phenotype analysis when available. As mentioned above, several CEBFs (i.e., ERF1, AtERF2 and ORA59) are known to regulate defense responses. However, their involvement in ABA response and the functions of other CEBFs have not been reported yet. Here, we present our results obtained with CEBFs, AtERF13 and RAP2.4L. AtERF13 was found to possess very high transcriptional activity in yeast (Figure 1B) and localized in the nucleus. Its expression was limited to the shoot meristem region and young emerging leaves (Figure 2B), implying that it may play a role in shoot growth or development. Consistent with this notion, AtERF13 OX lines exhibited minor dwarfism (Figure 3A). The growth retardation observed in the OX lines may reflect the normal inhibitory role of AtERF13 or be the result of its ectopic overexpression. However, we think that AtERF13 probably play a role in growth regulation. Because we could not obtain its knockout lines, we prepared and analyzed its RNAi lines. Our results showed that the RNAi lines grew faster than wild type plants (Additional file 3), suggesting that AtERF13 may inhibit seedling growth.

Overexpression of AtERF13 conferred ABA hypersensitivity during postgermination growth. As shown in Figure 3 both shoot and root growth was severely inhibited by the low concentration of ABA, which had little effect on wild type seedling growth. Additionally, the AtERF13 OX lines were hypersensitive to glucose, whose effect is mediated by ABA. We did not carry out extensive expression analysis of ABA-responsive genes in AtERF13 OX lines. However, our limited target gene analysis showed that expression of several ABA-responsive genes was affected by AtERF13 (Figure 5). Thus, our results strongly suggest that AtERF13 may be involved in ABA response. As mentioned in the Results, we did not observe distinct phenotypes with AtERF13 RNAi lines except faster seedling growth, presumably because of the functional redundancy among CEBFs.

In the case of RAP2.4L, we did not observe changes in ABA sensitivity in its OX lines, although we observed up-regulation of several ABA-responsive genes (Figure 5). However, the transgenic lines were glucose-hypersensitive, suggesting that it may be involved in sugar response (Figure 4B). We also analyzed its knockout lines, but did not observe distinct phenotypes (not shown). RAP2.4 is the closest homologue of RAP2.4L; therefore, we also analyzed its OX and knockout phenotypes. We did not observe alterations in ABA response in either the OX or the knockout lines of RAP2.4 (not shown). The results are consistent with those observed by Lin et al. [26], who reported that RAP2.4 is involved in light, ethylene and ABA-independent drought tolerance but not in ABA response. However, similar to RAP2.4L OX lines, RAP2.4 OX lines were glucose-sensitive and both RAP2.4 and RAP2.4L OX lines were salt-sensitive (Figure 4E-4G). Additionally, single or double knockout lines of RAP2.4 and RAP2.4L grew faster than wild type plants (Additional file 3), suggesting their role in seedling growth control.

It is not known whether other CEBFs are involved in ABA response. Another important question to be addressed is the mechanism of their function, if they are involved in ABA response. CE1 constitutes an ABA response complex with the G-box type ABRE and functions in combination with ABRE. Therefore, CEBFs are expected to interact with the transcription factors ABFs/AREBs, which mediate ABA response in seedlings via the G-box type ABRE. In the case of DREB2C, which binds another coupling element DRE, its physical interaction with ABFs/AREBs has been demonstrated [37]. It would be worthwhile to determine whether CEBFs can physically interact with ABFs/AREBs. As described before, several CEBFs mediate plant defense response. Thus, our results raise an interesting possibility that CE1 may be a converging point of ABA and defense responses.

## Conclusions

We conducted one-hybrid screen to isolate proteins that interact with the coupling element CE1 and isolated a group of AP2/ERF superfamily proteins designated as CEBFs. To determine the function of CEBFs, we examined their expression patterns and prepared OX lines for phenotype analysis. Our results showed that the AtERF13 OX lines are ABA-and glucose-hypersensitive. The OX lines of two other CEBFs, RPA2.4 and RAP2.4L, were glucose-hypersensitive. Thus, overexpression of the three CEBFs resulted in alterations in ABA and/or sugar response. In addition, several ABA-regulated genes were up-regulated in the transgenic lines. Taken together, our data strongly suggest that the three CEBFs evaluated in this study are involved in ABA or stress response. The functions of other CEBFs remain to be determined.

## Methods

### One-hybrid screen

One-hybrid screen was conducted as described before [10]. To prepare reporter gene constructs, a trimer of the oligonucleotides, 5'-CATTGCCACCGGCCC-3', and its complementary oligonucleotides were annealed and cloned into the Zero Blunt TOPO (Invitrogen) vector. The insert was then excised out by Spe I-Eco RV or Kpn I-Xho I digestion. The fragments were then cloned into pSK1, which was prepared by Bam HI digestion followed by Klenow treatment and Spe I digestion, and Kpn I-Xho I digested pYC7-I, respectively. The reporter constructs were sequentially introduced into YPH500 to prepare reporter yeast harboring *HIS3/lacZ *double reporters. Yeast transformation was carried out as described before [10], using the cDNA library DNA representing mRNA isolated from Arabidopsis seedlings treated with ABA and salt.

Approximately 3.6 million yeast transformants were screened, and 78 positive clones were isolated. The positive clones were grouped according to the restriction patterns after EcoR1 and/or Hae III digestion of the insert DNA, which was prepared by PCR. Plasmid DNA was rescued from the representative clones of each group and other non-grouped clones and sequenced. Fifty two positive clones were analyzed and sequenced. For the confirmation test shown in Figure 1A, plasmid DNA from the positive clones was re-introduced into the yeast reporter strain and activation of the *lac*Z reporter was examined by a filter lift assay.

### Transcriptional assay

Transcriptional activity was determined employing a yeast system, as described previously [20]. The coding regions of CEBFs were prepared by PCR and individually cloned into the Sma I-Not I sites of pPC62/LexA bait vector containing *Lex*A DB. Primer sequences are available upon request. The bait constructs were subsequently introduced into L40 (MATα, his3Δ200, trp1-901, leu2-3112, ade2, LYS2::[LexAop(x4)-HIS3], URA3::LexAop(x8)-LacZ, GAL4) (Invitrogen, USA), which carries a *lac*Z reporter gene with *Lex*A binding sites in its promoter. β-galactosidase activity was measured by liquid assay using ONPG (O-nitrophenyl-*β*-D-galactopyranoside) as a substrate and expressed in Miller units.

### RNA isolation and expression analysis

RNA was isolated employing the RNeasy plant mini kit (Qiagen, USA). Northern blot analysis was carried out, as described previously [20]. For RT-PCR analysis, RNA was treated with DNase I to remove possible contaminating DNA before cDNA synthesis, and the first strand cDNA was synthesized using Superscript III (Invitrogen) according to the manufacturer's instructions. cDNA amplification was carried out within a linear range using gene-specific primers. For quantitative RT-PCR, the cDNA was diluted 10-fold, and PCR was performed with SsoFast EvaGreen supermix in a Bio-Rad CFX96 Real-Time PCR Systems (Bio-Rad) according to the supplier's instructions. Quantitation was carried out using the CFX96 Real-Time PCR Systems software, employing actin-1 as a reference gene. Primer sequences are available upon request.

### Determination of promoter activity and subcellular localization

To prepare promoter-GUS constructs, approximately 2.5 kb 5' flanking sequences of AtERF13, RAP2.4 and RAP2.4L were prepared and cloned into pBI101.2 [22]. For AtERF13, the promoter fragment was amplified from genomic DNA using the primer set 5'-AAG CTT GGT ACT AGT ACT GCT AGG TTT CTC-3' and 5'-AAT GGA TTC TTG AAT GCT TCT GAA-3'. The resulting fragment was digested with Hind III and then ligated with PBI101.2, which was predigested with Hand III and Sma I. For RAP2.4 and RAP2.4L, the primer sets, 5'-acg cgtc gac CAT CCC TGT ACC ACT CAC TAT CTT ATT C -3' and 5'-GAA TCC GAA AAA ATT GAA CCT GAG AC-3', and 5'-acg cgt cga cTA ACA CAC AAA ATG TAC CGA AAG AAG-3' and 5'-CTG TGT AGA TTT CTG AGA GGA GGG A-3' were employed to amplify the promoter fragments. The PCR products were then digested with Sal I and ligated with pBI101.2 cut with Sal I-Sma I. Transformation of Arabidopsis plants (Ecotype, Landsberg *erecta*, L*er*) were according to Bechtold and Pelletier [40]. Histochemical GUS staining was conducted as describe before [41], using T2 or T3 generation plants.

To investigate the subcellular localization, the coding regions of AtERF13, RAP2.4 and RAP2.4L were fused with the EYFP coding region of p35S-FAST/EYFP in frame. The coding region of AtERF13 was prepared by PCR using the primers 5'-aag ccc ggg ATG AGC TCA TCT GAT TCC GTT AAT-3' and 5'- aag ccc ggg TAT CCG ATT ATC AGA ATA AGA ACA TT-3', and the amplified fragment was digested with Xma I prior to ligation with Xma I-cut p35S-FAST/EYFP. The coding region of RAP2.4 was amplified using the primer set 5'-aag gag ctc ATG GCA GCT GCT ATG AAT TTG TAC-3' and 5'- aag ccc ggg AGC TAG AAT CGA ATC CCA ATC GAT-3', whereas the coding region of RAP2.4L was amplified using the primers 5'- aag gag ctc ATG ACA ACT TCT ATG GAT TTT TAC AG-3' and 5'-aag ccc ggg ATT TAC AAG ACT CGA ACA CTG AAG-3'. The amplified fragments were treated with Sac I and Sma I and subsequently ligated with p35S-FAST/EYFP digested with the same enzymes.

Agrobacterium infiltration was according to Witt et al. and Voinnet et al. [42,43]. Tobacco (*Nicotiana benthamiana*) leaves were co-infiltrated with the *Agrobacterium strains *(C58C1) containing the above constructs and p19, respectively. The images of the tobacco epidermal cells were taken with the Olympus BX51 microscope with a YFP filter 40 hr after infiltration.

### Generation of transgenic plants and phenotype analysis

To prepare OX vector constructs, the coding regions of AtERF13, RAP2.4 and RAP2.4L were amplified from a cDNA library and cloned into pBI121. The RAP2.4 coding region was amplified using the primers 5'- TAG GAT CCA TGG CAG CTG CTA TGA ATT TGT ACA CTT G-3' and 5'- TTG CCC CTA AGC TAG AAT CGA ATC CCA ATC-3'. The RAP2.4L coding region was amplified using the primers 5'- CCG GAT CCA TGA CAA CTT CTA TGG ATT TTT ACA GT-3' and 5'- CAA CAT CTA ATT TAC AAG ACT CGA ACA CT-3'. The amplified fragments were digested with Bam HI and cloned into pBI121, which was prepared by the removal of the GUS coding region after Bam HI-Eco ICRI digestion. The coding region of AtERF13 was prepared by PCR using the primer set 5'- CGT CTA GAA TGA GCT CAT CTG ATT CCG TTA ATA ACG G-3' and 5'- AAC TAA TTA TAT CCG ATT ATC AGA ATA AG-3'. The fragment was treated with Xba I and ligated with GUS-less pBI121, which was prepared by removal of the GUS coding region after Xba I-Eco ICRI digestion.

For the AtERF13 RNAi construct, the primers 5'-GGG GCG CGC CGC ATT TGA TTG GTT CTT GTA AGT ATG AG-3' and 5'- CGT AAA TTT ATA CTA TGG AAC CGA ATT TAG AAG-3' were used to amplify the 387 bp sense orientation fragment. The fragments were cloned into pFGC5941 after Asc I-Swa I digestion. Primers 5'- GGT CTA GAG CAT TTG ATT GGT TCT TGT AAG TAT GAG-3' and 5'- CGG GAT CCT ACT ATG GAA CCG AAT TTA GAA G-3' were employed to amplify the antisense fragment, which was cloned into pFGC5941 containing the sense fragment after Bam HI-Xba I digestion. The intactness of the cloned sequences of all of the constructs used in this study was confirmed by DNA sequencing.

Arabidopsis transformation was carried out as described above. More than ten transgenic lines were recovered for each CEBF, and T3 or T4 generation homozygous lines were employed for phenotype analysis, which was carried out as described before [41].

Seeds of knockout lines, SALK_093377 and SALK_091654 for RAP2.4 and RAP2.4L, respectively, were obtained from the Arabidopsis stock center. In the case of SALK_093377, homozygous knockout sublines were recovered from the plants whose progeny segregated with 3:1 ratio of kanamycin resistance and kanamycin susceptible seeds. In the case of SALK_091654, the plants were susceptible to kanamycin, and homozygous knockout sublines were recovered after genomic PCR of individual plants. Insertion of the T-DNA into the annotated position was confirmed by genomic PCR and sequencing of the amplified fragments.

## Authors' contributions

SL conducted the expression analysis and analyzed the OX and KO lines. JHP conducted yeast one-hybrid screens. MHL and JY prepared OX lines and analyzed their phenotypes. SYK designed experiments and wrote the paper. All authors read and approved the final manuscript.

## Supplementary Material

Additional file 1 **Salt tolerance of AtERF13 OX lines**. Plants were germinated and grown on MS medium containing 75 mM or 125 mM NaCl for 10 days before photographs were taken. Click here for file

Additional file 2 **Mannitol response of RAP2.4L and RAP2.4 OX lines**. Plants were germinated and grown on MS medium containing 4% mannitol for 13 days before photographs were taken. R, RAP2.4 OX lines. RL, RAP2.4L OX lines. L*er*, Landsberg *erecta*. Click here for file

Additional file 3 **Growth of AtERF13, RAP2.4 and RAP2.4L knockout and RNAi lines**. RNAi lines of AtERF13 and knockout lines of RAP2.4 and RAP2.4L were prepared as described in the Methods, and their growth phenotypes were investigated. (A) RNAi lines of AtERF13. Top, AtERF13 expression levels determined by RT-PCR. RNA was isolated from plants grown under normal condition. Bottom, plants grown in soil for 25 days. #25 and #31 denote RNAi lines. (B) Single or double knockout (KO) lines of RAP2.4 and RAP2.4L. Top left, expression levels of RAP2.4 and RAP2.4L in the single knockout lines of RAP2.4 (RK) and RAP2.4L (RLK) determined by RT-PCR. Top right, expression levels of RAP2.4 and RAP2.4L in the double knockout line (DK). Bottom, plants grown in soil for four weeks. Click here for file
