# Supplementary figures and images for: Isolation and functional characterization of CE1 binding proteins
Source: BMC Plant Biol. 2010 Dec 16;10:277. doi: 10.1186/1471-2229-10-277 (PMC3016407; doi:10.1186/1471-2229-10-277)

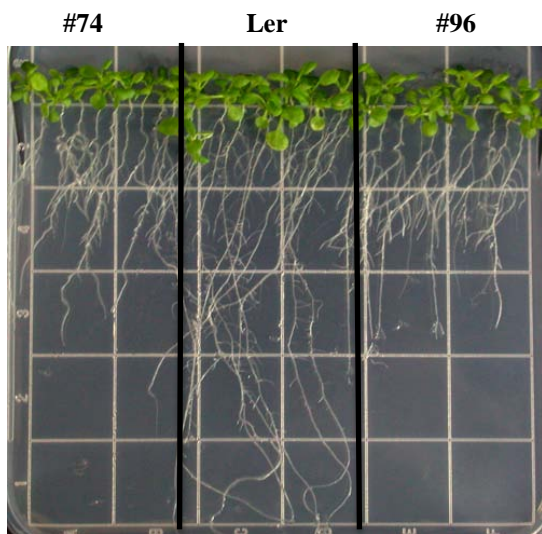

MS

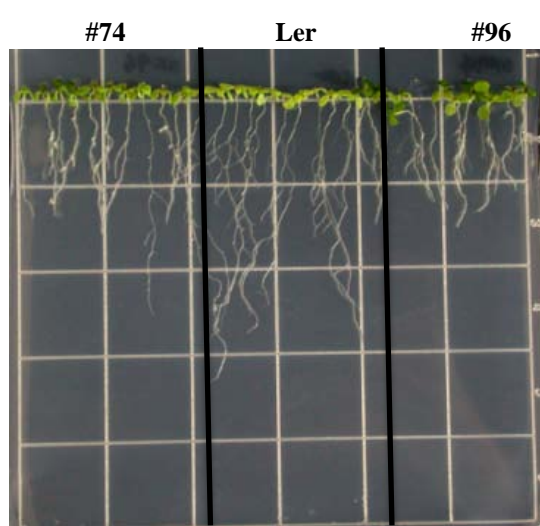

Salt 75mM

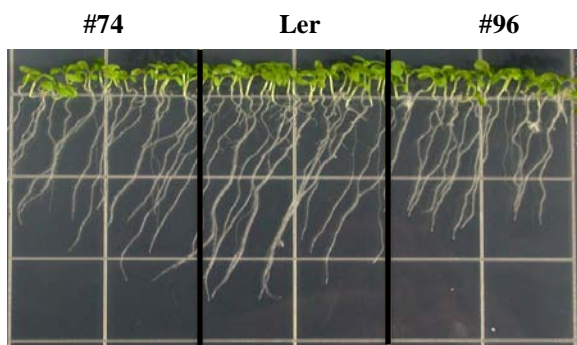

MS

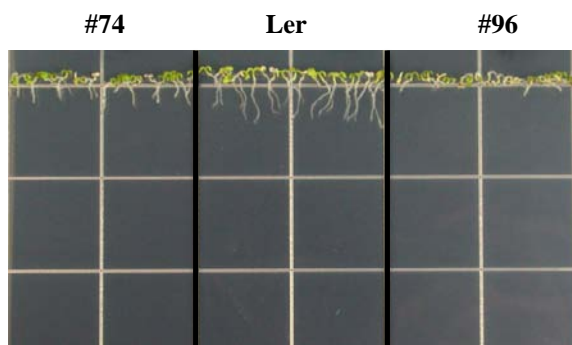

Salt 125mM

Supplement: Additional file 1 — Salt tolerance of AtERF13 OX lines. Plants were germinated and grown on MS medium containing 75 mM or 125 mM NaCl for 10 days before photographs were taken. [file 1471-2229-10-277-S1.PDF]

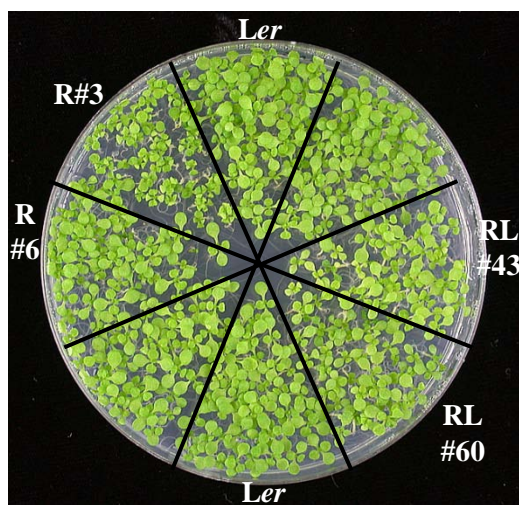

**MS**

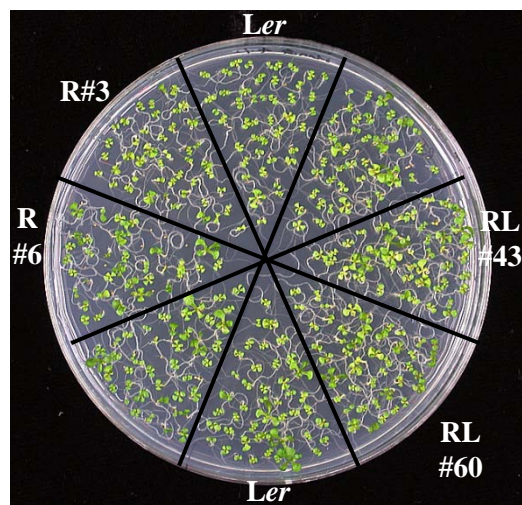

**4% Mannitol**

Supplement: Additional file 2 — Mannitol response of RAP2.4L and RAP2.4 OX lines. Plants were germinated and grown on MS medium containing 4% mannitol for 13 days before photographs were taken. R, RAP2.4 OX lines. RL, RAP2.4L OX lines. Ler, Landsberg erecta. [file 1471-2229-10-277-S2.PDF]

**A**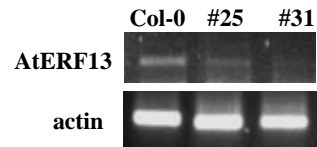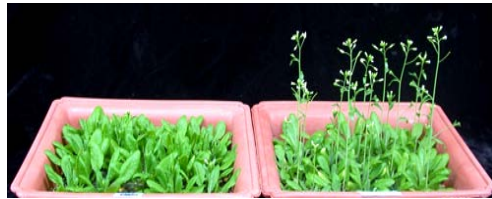**Col-0****#25****B**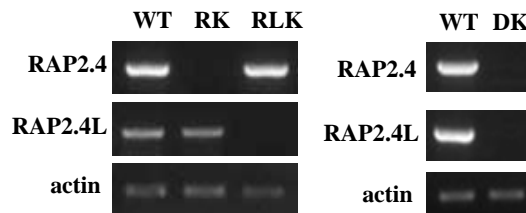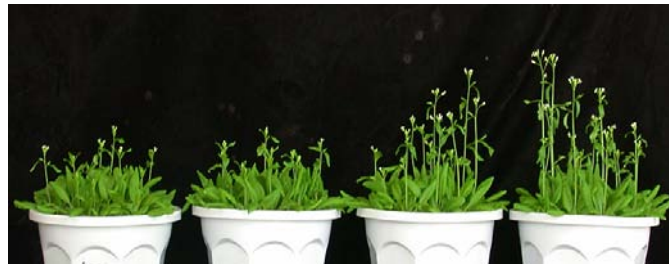**Col-0  
(WT)****RAP2.4  
KO****RAP2.4L  
KO****Double KO**

Supplement: Additional file 3 — Growth of AtERF13, RAP2.4 and RAP2.4L knockout and RNAi lines. RNAi lines of AtERF13 and knockout lines of RAP2.4 and RAP2.4L were prepared as described in the Methods, and their growth phenotypes were investigated. (A) RNAi lines of AtERF13. Top, AtERF13 expression levels determined by RT-PCR. RNA was isolated from plants grown under normal condition. Bottom, plants grown in soil for 25 days. #25 and #31 denote RNAi lines. (B) Single or double knockout (KO) lines of RAP2.4 and RAP2.4L. Top left, expression levels of RAP2.4 and RAP2.4L in the single knockout lines of RAP2.4 (RK) and RAP2.4L (RLK) determined by RT-PCR. Top right, expression levels of RAP2.4 and RAP2.4L in the double knockout line (DK). Bottom, plants grown in soil for four weeks. [file 1471-2229-10-277-S3.PDF]
